# Opioid prescribing among aged care residents during the first year of the COVID-19 pandemic: an analysis using general practice health records in Australia

**DOI:** 10.1186/s12877-023-03821-5

**Published:** 2023-02-24

**Authors:** Zhaoli Dai, Magdalena Z. Raban, Gorkem Sezgin, Precious McGuire, Shirmilla Datta, Nasir Wabe, Christopher Pearce, Richard Woodman, Andrew Georgiou

**Affiliations:** 1 Centre for Health Systems and Safety Research, Australian Institute of Health Innovation, Faculty of Medicine, Health and Human Sciences, Macquarie University, Sydney, NSW 2109 Australia; 2 Health Sciences Building, College of Medicine and Public, Health Flinders University, Sturt Road, Bedford Park, Adelaide, SA 5042 Australia; 3 Eastern Melbourne Primary Health Network, Victoria, Australia

**Keywords:** Opioids, Aged care, Nursing homes, Pain management, General practice, Primary care, COVID-19

## Abstract

**Background:**

Opioid use is common among adults 65 years and older, while long-term use of opioids remains controversial and poses risks of drug dependence and other adverse events. The acute disease caused by the SARS-CoV-2 (COVID-19) pandemic has created new challenges and barriers to healthcare access, particularly for long-term care residents. Australia had a relatively low incidence and deaths due to COVID-19 during the first year of the pandemic compared to most OECD countries. In this context, we examined opioid prescribing rates and their dosage in residential aged care facilities (RACFs) before (2019) and during the COVID-19 pandemic (2020) from March to December in Australia.

**Methods:**

We conducted a retrospective cohort analysis using general practice electronic health records. This includes 17,304 RACF residents aged 65 years and over from 361 general practices in New South Wales and Victoria. Number of opioid prescriptions and percentage of opioids over 50 mg/day of oral morphine equivalent (OME) were described. Multivariate generalized estimating equations were applied to estimate odds ratios [aORs (95% confidence intervals)] for 1) opioids prescribed per consultation and 2) prescription opioids over 50 mg/day OME.

**Results:**

In 2020 among 11,154 residents, 22.8% of 90,897 total prescriptions were opioids, and of the opioids, 11.3% were over 50 mg/day OME. In 2019 among 10,506 residents, 18.8% of 71,829 total prescriptions were opioids, of which 10.3% were over 50 mg/day OME. Year [2020 vs. 2019: aOR (95% CI):1.50 (1.44, 1.56); 1.29 (1.15, 1.46)] and regionality [rural/regional vs. metropolitan: 1.37 (1.26, 1.49); 1.40 (1.14, 1.71)] were associated with higher odds of prescription opioids and OME > 50 mg/day, respectively. Similar results were found when limited to the same residents (*n* = 7,340) recorded in both years.

**Conclusions:**

Higher prescription rates of opioids were observed during the COVID-19 pandemic in 2020 than in 2019 in Australian RACFs. The higher odds of prescription opioids and higher dosing in rural/regional than metropolitan areas indicate a widening of the gap in the quality of pain management during the pandemic. Our findings contribute to the limited data that indicate increased opioid prescriptions in long-term care facilities, which is likely to continue while COVID-19 pandemic restrictions remain.

**Supplementary Information:**

The online version contains supplementary material available at 10.1186/s12877-023-03821-5.

## Introduction

Chronic pain is a common condition in older adults, with up to 80% of long-term care residents experiencing this condition [1]. In 2020, the Australian Institute of Health and Welfare reported that opioids were dispensed to treat chronic pain in 40% of adults 45 years and older, making them the most common analgesics used to manage pain [2]. Opioid use is also common in residential aged care facilities (RACFs) in Australia, with about half of the general practice consultations involving discussion on pain management [3–5]. Consistent with this, the global data have suggested that the prevalence of opioid use ranges from 22.4% to 51.7% in adults 65 years and older in a systematic review [6]. However, despite its prevalence in older adults, long-term use of opioids for chronic non-cancer pain remains controversial and poses risks of drug dependence, falls, hospitalization, and mortality in this population [7]. Hence, appropriate prescribing and use of opioids requires regular monitoring.

The acute disease caused by the SARS-CoV-2 (COVID-19) pandemic has created new challenges and barriers to healthcare access, particularly for residents in long-term care facilities. Before the pandemic, about half of the general practice consultations involved discussion on pain management [3–5]. Due to the multiple lockdowns and a prolonged period of isolation during the pandemic, general practitioners’ (GPs) in-person visits to residential aged care facilities (RACFs) decreased, particularly around the second wave in Victoria [8]. This may trigger a higher incidence of chronic pain symptoms in residents in RACFs, because of increased anxiety, psychosocial stress, depression, and loneliness [9], with fewer routine physician or family visits, potentially leading to a higher demand for pain medications.

While prescribed opioids are likely different during the pandemic due to increased reported pain, the global pharmaceutical sales data has suggested a downward trend in opioid use between 2015 and 2019 in Australia [10] and decreased prescribed opioids over 20 oral morphine milligram equivalent (OME) from 2015 to 2018 in Queensland [11]. On the other hand, general practitioner (GP) in-person visits to RACFs also decreased during the pandemic, with an increase in telehealth service utilization [12], particularly around the second wave (June to October 2020) in Victoria. Considering all of these changes during the pandemic, there is limited data on prescribed opioids in long-term care settings in the Asia–Pacific region.

Optimal pain management is a vital metric for the quality of care in RACFs [13]. This requires understanding how opioids are prescribed under different circumstances. Hence, this study compared opioid prescriptions before and during the pandemic between 2019 and 2020 in RACFs and examined how sociodemographic characteristics and general practice care delivery modes affected prescribed opioids.

## Methods

### Data source

We used extracted data from general practice electronic health records stored in a secure digital health platform, Population Level Analysis & Reporting (POLAR), to collate and identify aged care residents. The POLAR dataset at the time of this study draws from 840 general practices in Victoria (*n* = 502) and New South Wales (*n* = 338), the two most populous states in Australia [14]. This data source captures de-identified patient records and general practice consultations, medication prescriptions, and pathology testing and has been used by researchers to generate evidence on population health [15–17].

The current study extracted data from persons residing in RACFs, involving 361 practices (139 for New South Wales and 222 for Victoria). We reported this study according to the items listed on the Reporting of studies Conducted using Observational Routinely Collected Data for observational studies using routinely collected health data [18].

### Study sample

We applied the following criteria to identify aged care residents, including 1) Medicare Benefits Schedule (MBS) billing items claimed specifically by GPs in residential aged care facilities (Supplementary Table 1); 2) persons aged 65 years or over, a general requirement for age-eligible in residential aged care facilities; and 3) persons who had received general practice services three or more times in the past two years, defined as active patients [19]. After merging with the medications data, 17,304 persons were identified for 2019–2020.

### Demographic characteristics

Age group (65–69 years, 70–74 years, 75–79 years, 80–84 years, and 85 years and above), sex (male, female), regionality (metropolitan, rural/regional areas), and state (New South Wales, Victoria) were included in the analysis. The reason to include state was that New South Wales (April – May 2020) and Victoria (June – October 2020) had differing COVID-19 outbreaks in 2020. In addition, socioeconomic status (SES) was adjusted as a confounder based on the postcode of an aged care facility but not their residential household.

### General practice standard consultations

Besides the MBS items mentioned above for face-to-face consultations, telehealth MBS items were included (Supplementary Table 1) to calculate the total counts of GP consultations and the proportion of telehealth consults.

### Opioid prescribing patterns

Prescribing patterns for all medications and opioid analgesics were charted for 2019 and 2020, presented as mean scripts per 100 persons per month. The distribution of opioids based on the Anatomical Therapeutic Chemical Classification System NO2A classification was also compared (buprenorphine, oxycodone, morphine, fentanyl, tramadol, codeine, and hydromorphone).

### Opioid prescribing status in regression analysis

Two binary outcomes were generated to assess: i) whether opioids were prescribed (Yes or No) to a resident during any consultation, and ii) whether daily doses of prescribed opioids exceeded 50 mg/day OME, using methods published in the Australian Medication Handbook [20]. To extract prescribed opioids from the electronic health records, we used the Anatomical Therapeutic Chemical (ATC) code for opioids, defined in POLAR, for each drug classification. Based on the generic name of a medication collected in POLAR, we further used the ATC Classification System NO2A (opioids) classification to define opioid types as buprenorphine, oxycodone, morphine, fentanyl, tramadol, codeine, and hydromorphone. To calculate OME for each opioid type, we multiplied milligrams of the total opioids intake per day using the medication details collected in POLAR, including dose, strength, and frequency, with the respective OME conversion factor. In sensitivity analyses, where data were missing on dose or frequency, we imputed OME for controlled-release opioids as these medications have standard dosing intervals. This resulted in imputing 22% (1,213/5,486) of the prescription opioids with missing doses or frequency.

### Statistical analysis

Descriptive statistics for both overall medications and prescribed opioid analgesics included the total, percentage increase, and mean rate (per 100 residents per month) with 95% confidence intervals calculated. In addition, differences in the mean rate were compared between the two years using an ANCOVA test, after adjusting for demographic factors.

We used univariate and multivariate generalized estimating equations (GEE) to estimate independent odds ratios (ORs) and 95% CIs for opioid prescribing status (yes/no) and for prescribed opioids > 50 mg/day OME. The multivariate models included year (2019, 2020), month (March to December), age group (65–69 y, 70–74 y, 75–79 y, 80–84 y, and 85 y +), sex (female, male), regionality (metropolitan and regional areas), SES, and State (New South Wales, NSW; Victoria). A robust sandwich estimator was applied to cluster standard errors for repeated measures within the residents and between the residents nested in the general practices.

We performed a sensitivity analysis restricting the sample to residents included in both 2019 and 2020. This was critical as it ensured that our results were not affected by any significant changes in the distribution of comorbidities, such as cancer, within the complete sample. Consistency in the results with the primary analysis using the complete cohort would indicate that any variability of opioid prescribing between years was not due to possible differences in the prevalence of comorbidities between the two periods. In further sensitivity analysis, we also included interactions to assess any differences in the demographic characteristics modified by year or State.

All analyses were conducted using Stata 16 MP (StataCorp, TX., USA). Statistical significance was set using two-sided alpha at 0.05.

## Results

### Sample selection

**Fig. 1:**
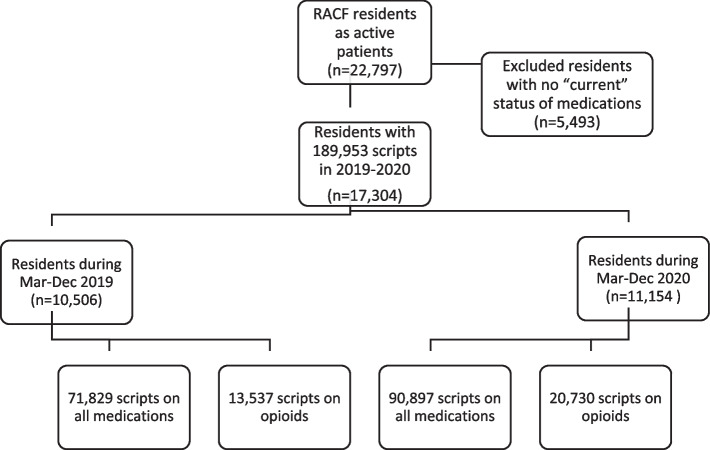
Selection of eligible study participants in the study

We included 17,304 eligible individuals in our analysis. Between March and December, there were 71,829 prescriptions on any medication and 13,537 prescriptions on opioids (18.8%, 13,537/71,829) among 10,506 residents in 2019 and 90,897 prescriptions on any medication and 20,730 opioid prescriptions (22.8%, 20,730/90,897) among 11,154 residents in 2020. Figure 1 describes the process for the sample selection.

### Characteristics of included participants

The demographic characteristics of the residents are described in Table 1. The majority of the residents aged 85 years and above (> 60%) were female (> 67%), resided in metropolitan areas (80%), and were residents of Victoria (74%). From March to December 2020, 17.6% of consultations were conducted by telephone and less than 2% by video.

**Table 1:** Characteristics of aged care residents and general practice consultations from March to December in 2019 and 2020

	**2019** ***n* = 10,506**	**2020** ***n* = 11,154**
**Age group**		
65—69y, n (%)	325 (3.1)	390 (3.5)
70—74y, n (%)	662 (6.3)	719 (6.5)
75—79y, n (%)	996 (9.5)	1129 (10.1)
80—84y, n (%)	1741 (16.6)	1917 (17.2)
85 + y, n (%)	6782 (64.5)	6999 (62.8)
**Sex**		
Male, n (%)	3357 (32.0)	3663 (32.8)
Female, n (%)	7149 (68.0)	7491 (67.2)
**Socioeconomic status**		
High, n (%)	5776 (55.0)	6031 (54.1)
Middle, n (%)	3163 (30.1)	3467 (31.1)
Low, n (%)	1567 (14.9)	1656 (14.9)
**State**		
NSW, n (%)	2741 (26.1)	2763 (24.8)
VIC, n (%)	7765 (73.9)	8391 (75.2)
**Regionality**		
Metropolitan, n (%)	8451 (80.4)	8884 (79.6)
Regional, n (%)	2055 (19.6)	2270 (20.4)
**Proportion of GP consultations**		
Face-to-face consultations (%)	100	81.0
Phone consultations (%)	0	17.6
Video consultations (%)	0	1.4

### Overall medications prescribing

Table 2 describes the number of prescriptions and the differences by year from March to December. We observed a 26.5% increase in the total number of prescriptions in 2020. Within this increase, opioids accounted for 22.8% of all medications in 2020 and 18.8% in 2019. In addition, the mean (95% CI) for the number of medication prescriptions per 100 persons per month was higher in 2020 than in 2019 [258 (255, 260) versus 233 (231, 236), respectively, *p* < 0.0001]. By contrast, the overall number of prescription opioids increased by 53.1% in 2020 compared to 2019 (20,730 versus 13,537, respectively). Similarly, the mean opioid prescriptions/100 persons/month were higher in 2020 than in 2019 [191 (189, 194) versus 182 (180, 185), respectively, *p* < 0.0001]. The results were similar (52.9% increase in total opioids) when restricted to the same residents (*n* = 7,340) appearing in both 2020 and 2019 (15,578 versus 10,190 total opioid prescriptions, respectively; *p* < 0.0001) and prescriptions/100 persons/month [198 (195, 201) versus 188 (185, 191), respectively; *p* < 0.001].

**Table 2:** Comparison of the number of prescriptions between 2019 and 2020 from March to December

Type of medication	Number of scripts per year		% Increase	Scripts per 100 persons per month (mean, 95% CI)		*p*-value* for the mean difference
	2019	2020		2019	2020	
Whole study sample (*n* = 10,506 person in 2019; *n* = 11,154 persons in 2020)						
Any type	71,829	90,897	26.5	233 (231, 236)	258 (255, 260)	*p* < 0.0001
Opioids	13,537	20,730	53.1	182 (180, 185)	191 (189, 194)	*p* < 0.0001
Opioids over 50 mg/day OME	1,399	2,346	67.7	174 (167, 182)	179 (173, 185)	p = 0.35
Sub-group of residents in both years (*n* = 7,340)						
Any type	53,870	61,984	15.1	226 (224, 229)	243 (240, 245)	*p* < 0.0001
Opioids	10,190	15,578	52.9	188 (185, 191)	198 (195, 201)	*p* < 0.0001
Opioids over 50 mg/day OME	1,071	1,478	38.0	185 (177, 193)	191 (183, 199)	*p* = 0.29

Statistical difference between the groups was calculated based on ANCOVA, after adjusting for Month (March to December), age group (65–69 y, 70–74 y, 75–79 y, 80–84 y, and 85 y +), sex (female, male), SES (low, middle, high), regionality (metropolitan and regional areas), and state (New South Wales, NSW; Victoria)

### Opioid prescribing

The types of opioids prescribed were comparable between 2019 and 2020, with buprenorphine (37.7% and 37.2%, respectively) and oxycodone (41.8% and 42.1%, respectively) being the primary opioids prescribed. Morphine, fentanyl, tramadol, and hydromorphone were similar between 2020 and 2019. Opioids over 50 mg/day OME accounted for 10.3% of prescription opioids in 2019 and 11.3% in 2020, with no difference being found for the mean rate of opioids over 50 mg/day OME per 100 persons per month between the years (Table 2). Furthermore, results were similar before and after the imputation or when restricted to the same residents with records in both years (*n* = 7,340).

### Demographic characteristics in association with opioid prescribing

Comparing 2020 with 2019, residents had 50% higher odds of receiving a prescription for opioids (aOR: 1.50; 95% CI:1.44–1.56) and 29% higher odds of being prescribed opioids above 50 mg/day OME (aOR: 1.29; 95% CI: 1.15–1.46; *p* < 0.001). Additionally, residents in rural/regional areas had 37% higher odds of receiving opioid prescriptions (aOR: 1.37; 95% CI: 1.26–1.49) and 40% higher odds of receiving prescribed 50 mg/day OME (aOR: 1.40; 95% CI: 1.14—1.71) compared to those living in metropolitan areas. Other predictors for prescribed opioids included age group, sex, and State (Table 3).

**Table 3:** Odds ratios (95% confidence intervals) for the association between demographic characteristics and opioid prescribing status in 2019–2020, March to December

**Characteristics**	**Prescribed** **any opioids**^**a**^	**Model 1**^**c**^	**Model 2 **^**d**^	**Characteristics**	**Opioid** **doses (OME** ** > 50 mg/day)**^**b**^	**Model 1**^**c**^	**Model 2 **^**d**^
	(%)				(%)		
**Year**				**Year**			
2019	21.8	Ref	Ref	2019	13.3	Ref	Ref
2020	27.0	1.46 (1.40, 1.52)	1.50 (1.44, 1.56)	2020	14.3	1.26 (1.13, 1.39)	1.29 (1.15, 1.46)
**Age group (years)**				**Age group (years)**			
65—69	21.6	Ref	Ref	65—69	21.3	Ref	Ref
70—74	22.7	1.10 (0.88, 1.36)	1.14 (0.92, 1.42)	70—74	26.2	1.09 (0.68, 1.72)	1.05 (0.66, 1.66)
75—79	24.0	1.15 (0.94, 1.41)	1.20 (0.98, 1.47)	75—79	21.7	1.01 (0.65, 1.58)	0.94 (0.61, 1.45)
80—84	22.9	1.06 (0.88, 1.28)	1.10 (0.90, 1.33)	80—84	16.7	0.75 (0.49, 1.16)	0.73 (0.48, 1.12)
85 +	25.5	1.17 (0.98, 1.40)	1.20 (1.00, 1.44)	85 +	10.7	0.52 (0.35, 0.77)	0.51 (0.34, 0.76)
**Sex**				**Sex**			
Female	26.4	Ref	Ref	Female	13.5	Ref	Ref
Male	20.7	0.82 (0.77, 0.88)	0.81 (0.76, 0 .87)	Male	15.0	1.10 (0.93, 1.31)	1.01 (0.84, 1.21)
**Regionality**				**Regionality**			
Metropolitan	23.8	Ref	Ref	Metropolitan	13.0	Ref	Ref
Regional	25.9	1.17 (1.09, 1.26)	1.37 (1.26, 1.49)	Regional	16.6	1.48 (1.24, 1.77)	1.40 (1.14, 1.71)
**State**							
NSW	34.8	Ref	Ref	NSW	13.8	Ref	Ref
Victoria	21.5	0.51 (0.47, 0.54)	0.48 (0.45, 0.52)	Victoria	14.0	1.38 (1.15, 1.66)	1.31 (1.08, 1.60)

^a^Analysis was based on 74,104 records, including residents with any medication records per month

^b^Analysis was based on 16,984 records, including residents with any opioid medication records per month

^c^Model 1, a univariate model without adjusting for any other covariates

^d^Model 2, a multivariate model including Year (2019, 2020), Month (March to December), age group (65–69 y, 70–74 y, 75–79 y, 80–84 y, and 85 y +), sex (female, male), SES (low, middle, high), regionality (metropolitan and regional areas), state (New South Wales, NSW; Victoria)

### Consultation mode in association with opioid prescribing

Residents who had face-to-face consultations were more likely to be prescribed opioids than those who did not have face-to-face consultations (aOR: 1.11; 95% CI: 1.03 -1.20) (Table 4). Similarly, residents that had both face-to-face and telehealth consultations were more likely to be prescribed opioids than those who had telehealth consultations only from March to December 2020 (adjusted OR: 1.17; 95% CI: 1.01—1.17) (Table 4). There were no associations between the type of consultation and prescribed opioids over 50 mg/day OME (Table 4).

**Table 4:** Odds ratios (95% confidence intervals) for the association between general practice consultation modes and opioid prescribing status in 2020, March to December

Consultation modes	Prescribed any opioids^a^	Odds ratio (95% CI)^c^	Odds ratio (95% CI)^d^	Consultation modes	Opioid doses over 50 mg/day OME^b^	Odds ratio (95% CI)^c^	Odds ratio (95% CI)^d^
	**%**				**%**		
Telehealth consultations only	23.6	Ref	Ref	Telehealth consultationsonly	18.6	Ref	Ref
Face-to-face consultations only	28.1	1.14 (1.06, 1.23)	1.11 (1.03, 1.20)	Face-to-face consultations only	13.3	1.06 (0.88, 1.28)	1.06 (0.88, 1.30)
Face-to-face and telehealth consultations	28.2	1.16 (1.08, 1.26)	1.17 (1.01, 1.17)	Face-to-face and telehealth consultations	16.4	1.03 (0.85, 1.24)	1.02 (0.85, 1.22)

^a^Analysis was based on 31,645 records for residents with any medication records

^b^Analysis was based on 8279 records for residents with any opioid medication records

^c^Model 1, a univariate model without adjusting for any covariates

^d^Model 2, a multivariate model after adjusting for Month (March to December), age group (65–69 y, 70–74 y, 75–79 y, 80–84 y, and 85 y +), sex (female, male), SES (low, middle, high), regionality (metropolitan and regional areas), state (New South Wales, NSW; Victoria), and RMMR (an MBS item billed for medication review in residential aged care facilities

### Sensitivity analysis

When limited to the same residents who had medication records in both 2019 and 2020, our regression analysis showed similar results as the overall analysis (Supplementary Table 2). Additionally, when stratified by year there was higher odds of prescribing opioids in rural/regional areas than in metropolitan areas in 2020 (aOR: 1.57; 95% CI:1.42—1.74) compared to 2019 (aOR: 1.24; 95% CI: 1.10—1.40) (Supplementary Table 3). Also, both states (NSW: aOR: 1.60; 95% CI: 1.49—1.71; Victoria: aOR: 1.45; 95% CI:1.38—1.53) showed higher odds of prescribed opioids in 2020 than in 2019 (Supplementary Table 4). Additionally, residents in rural/regional were more likely to receive opioid prescriptions in New South Wales (aOR: 2.36; 95% CI: 1.99—2.79) but not in Victoria (Supplemental Table 4). Finally, while there were no significant interactions between either year, or State and the demographic characteristics for opioid doses over 50 mg/day OME, residents in rural/regional areas had higher odds of these high doses of opioids than those in metropolitan areas [aOR (95% CI) for 2019: 1.36 (1.02–1.81); for 2020: 1.43 (1.13–1.64); for Victoria including 2019–2020: 1.30 (1.10 – 1.54); and for NSW including 2019–2020: 1.33 (1.05–1.67)].

## Discussion

In this retrospective cohort study of RACF residents in Australia, the absolute number of prescribed opioids increased by more than 50% during the COVID-19 pandemic in 2020 compared with the same period in 2019, and the odds of being prescribed an opioid analgesic increased by 50%. In addition, we found higher odds of being prescribed opioids above 50 mg/day OME in 2020 than in 2019, although these prescriptions per 100 persons per month were similar between 2019 and 2020. Our results also suggest that residents living in rural/regional aged care facilities were more likely to receive opioid prescriptions than those in metropolitan areas, with this association being more apparent in 2020 than in 2019.

Our results suggest that the COVID-19 pandemic is associated with a significant increase in prescribed opioids, evident in both the complete cohort in 2019–2020 (191 versus 182 opioid prescriptions per 100 persons per month) and among the sub-cohort of residents in both years (198 versus 188 opioid prescriptions per 100 persons per month). Consistent with our findings, a Canadian study also found a significant increase in prescribed opioids between the observed and projected use in the last week of September 2020 in nursing home residents during the COVID-19 pandemic [21]. Another study in the UK also found a higher proportion of patients being prescribed opioids during the COVID-19 pandemic than in the same period before the pandemic. In contrast, the use of strong opioids was similar before and during the COVID-19 pandemic [22]. This is similar to what we found in this study. Taken together, these data suggest that opioid prescriptions were greater during the COVID-19 pandemic in 2020 than prior to the pandemic found in studies across different countries.

Such increased opioid prescribing patterns during the pandemic may be attributable to several factors. During the first year of the pandemic, residents may have experienced pain exacerbation due to the multiple lockdowns and isolations, leading to decreased access to exercise classes, less frequent visits by allied health professionals, and limited implementation of pain management programs [9]. In addition, the pandemic may also affect overall medication stock and supply, resulting in fluctuations in prescribing patterns of different medicines. In addition to the circumstances due to the pandemic, the Australian Therapeutic Goods Administration implemented several restrictions on opioid prescribing in 2020, focusing on the safety and necessary use of opioid analgesics, including reduced pack sizes and unnecessary prescription of potent opioids such as fentanyl. This policy could have also affected opioid prescribing patterns, with our data underestimating the actual increases.

Our findings from multiple analyses on the higher odds of opioid prescribing amongst rural/regional RACFs than in metropolitan areas raise several concerns. Along with the data reported in the 2018 Australian national report on the higher frequency of opioids (1.4 fold) and higher OMEs (1.7 fold) dispensed in the inner regional than metropolitan areas [23], our study highlights a potentially widened gap in health disparities in these regions during the pandemic, where there were likely higher disease burdens of chronic pain, musculoskeletal disorders, and mental health disorders [24], limited healthcare resources (e.g., a lack of GPs and allied health professionals) and fewer opportunities of specialist referrals for pain management [25]. These discrepancies are presumably amplified during the pandemic.

Another unique finding in this study is that it incorporated the role of telehealth in opioid prescription. Our findings suggest that GPs were more likely to prescribe opioids following a face-to-face consultation. This is in line with a previous review by Mikelyte and colleagues, who found that prescribers’ frequent visits to patients were linked to safer opioid prescribing decisions for older adults [25]. Over 90% of the telehealth consultations were phone consults in this study, limiting our ability to assess how video (virtual) care played a role in opioid prescription. Therefore, future studies should examine the utilization of video consultations in pain management, especially in areas where resources and experienced GPs are limited.

### Strength and limitations

This study comprehensively reports opioid prescribing in residential aged care facilities during the first year of the COVID-19 pandemic in Australia. Major strengths include utilizing population-based linked health records in general practice and then applying stringent criteria to identify aged care residents, which provided a sufficiently large sample size to assess differences in opioid prescriptions before and during the first year of the COVID-19 pandemic. In addition, although many restrictions have been lifted in the communities since 2022, protective measures remain similar, with high levels of infectious disease control measures still existing in long-term care facilities for residents, care workers, physicians, and visitors. Hence, our findings on prescribed opioids in aged care facilities during the first year of the pandemic are clinically meaningful and relevant as people today are still living in the COVID-19 pandemic in many countries.

A major limitation of the study was the inability to assess residents’ health status and diagnosis due to a lack of access to linkage to hospitals and ascertain clinical diagnoses such as cancer and other acute conditions that require opioid use. Although opioid adherence was not measured in the current analysis, we can reasonably assume that opioid use among these RACF residents during 2019–2020 is consistent, as evidenced by our sensitivity analysis among the subgroup of residents assessed in 2019–2020, which provides similar results to that for the whole cohort. This suggests that it is unlikely that changes in the prevalence of the underlying distribution of comorbidities or cancer between the two periods were a contributing factor.

Given the large sample size, it is likely that the overall demographics for the two periods were stable. Therefore, any abrupt change in physical conditions contributing to the increased demand for prescribed opioids is unlikely. In addition, while our study did not include opioids prescribed outside general practice, GPs are often the primary prescribers of opioids in aged care facilities. Finally, the generalisability of the results may be limited to countries with similar healthcare systems and COVID-19 restrictions.

### Conclusion and implications

During the first year of the COVID-19 pandemic in 2020, we found higher opioid prescription rates but not higher dosing of opioids over 50 mg/day OME in Australian residential aged care facilities. Notably, the independently increased odds of opioid prescriptions as well as higher opioid dosing in rural/regional facilities, indicate a potentially increased widening of the gap in the quality of pain management in underserved communities, where limited care access and health disparities may have been amplified during the pandemic. Finally, our findings contribute to the limited data that indicate increased opioid prescriptions in long-term care facilities during the pandemic, which is likely to continue while the COVID-19 pandemic protective measures remain.

## Supplementary Information

**Additional file 1: Supplementary Table 1. **General practice standard consultation - Medicare Benefits Schedule (MBS) billing items. **Supplementary Table 2.** Association between demographic characteristics and opioid prescribing status from March to December 2019 - 2020 among the same residents (*n*=7,340) recorded in both years. **Supplementary Table 3. **Stratified analyses for the association between demographic characteristics and opioid prescribing status between 2019 and 2020, March to December. **Supplementary Table 4. **Stratified analyses for the association between demographic characteristics and opioid prescribing status between the state of New South Wales (NSW) and Victoria from March to December, 2019-2020.

## Data Availability

The data supporting this study’s findings are available from the data custodian, Outcome Health. However, restrictions apply to the availability of these data, which were used under license for the current study and are not publicly available. Data are, however, available from the authors upon reasonable request (Email to Zhaoli Dai, zhaoli.daikeller@flinders.edu.au) and with the permission of Outcome Health.
