# Supplementary material for: Opioid prescribing among aged care residents during the first year of the COVID-19 pandemic: an analysis using general practice health records in Australia
Source: BMC Geriatr. 2023 Feb 24;23:111. doi: 10.1186/s12877-023-03821-5 (PMC9950695; doi:10.1186/s12877-023-03821-5)
Supplement: Supplementary file 1 — Additional file 1: Supplementary Table 1. General practice standard consultation - Medicare Benefits Schedule (MBS) billing items. Supplementary Table 2. Association between demographic characteristics and opioid prescribing status from March to December 2019 - 2020 among the same residents (n=7,340) recorded in both years. Supplementary Table 3. Stratified analyses for the association between demographic characteristics and opioid prescribing status between 2019 and 2020, March to December. Supplementary Table 4. Stratified analyses for the association between demographic characteristics and opioid prescribing status between the state of New South Wales (NSW) and Victoria from March to December, 2019-2020. [file 12877_2023_3821_MOESM1_ESM.docx]

**Dai et al. Opioid prescribing among aged care residents during the first year of the COVID-19 pandemic: An analysis using general practice health records in Australia**

**Supplementary materials**

| **Supplementary Table 1.** General practice standard consultation - Medicare Benefits Schedule (MBS) billing items | |
| --- | --- |
|  |  |
| **Type of consultations** | **MBS items** |
| Face-to-face[1] |  |
| Routine hours^1^ | 90020, 90035, 90043, 90051 |
| After hours^2^ | 5010, 5028, 5049, 5067 |
|  |  |
| Telehealth^3^[2] | |
| Telephone | 91809, 91810, 91795, 91811 |
| Videoconferencing | 91800, 91801, 91790, 91802 |

^1^ Applicable if the patient is a care recipient in a residential aged care facility; each code represents the duration of a consultation.

^2^ After-hours non-urgent items, including patients in residential aged care facility locations; each code represents a consultation duration.

^3^ Standard GP attendance items for telehealth; each code represents the duration of a consultation.

**Supplementary Table 2.** Association between demographic characteristics and opioid prescribing status from March to December 2019 - 2020 among the same residents (n=7,340) recorded in both years

| **Characteristics** | **Prescribed**  **any opioids^1^** | **Odds ratio**  **(95% CI)** |  | **Opioid doses (OME >50mg/**  **day)^2^** | **Odds ratio**  **(95% CI)** |  |
| --- | --- | --- | --- | --- | --- | --- |
|  | (%) |  |  | (%) |  |  |
| **Year** |  |  |  |  |  |  |
| 2019 | 19.9 | Ref |  | 13.4 | Ref | |
| 2020 | 27.6 | 1.55 (1.48, 1.62) |  | 15.9 | 1.33 (1.15, 1.55) | |
|  |  |  |  |  |  | |
| **Age group (years)** |  |  |  |  |  | |
| 65 - 69 | 24.5 | Ref |  | 19.9 | Ref | |
| 70 - 74 | 23.2 | 0.95 (0.75, 1.20) |  | 34.1 | 1.04 (0.44, 2.49) | |
| 75 - 79 | 24.0 | 0.98 (0.79, 1.22) |  | 26.4 | 0.87 (0.37, 2.04) | |
| 80 - 84 | 22.3 | 0.90 (0.73, 1.11) |  | 19.4 | 0.72 (0.32, 1.63) | |
| 85+ | 24.1 | 0.97 (0.80, 1.18) |  | 10.6 | 0.38 (0.18, 0.81) | |
| **Sex** |  |  |  |  |  |  |
| Female | 25.0 | Ref |  | 14.2 | Ref |  |
| Male | 20.9 | 0.78 (0.73, 0.85) |  | 17.1 | 1.22 (0.85, 1.74) |  |
| **Regionality** |  |  |  |  |  |  |
| Metropolitan | 22.5 | Ref |  | 14.1 | Ref |  |
| Regional/rural | 28.0 | 1.43 (1.30, 1.56) |  | 16.6 | 1.44 (0.97, 2.16) |  |
| **State** |  |  |  |  |  |  |
| NSW | 28.0 | Ref |  | 15.7 | Ref |  |
| Victoria | 22.5 | 0.47 (0.37, 0.60) |  | 14.3 | 0.89 (0.62, 1.29) |  |

^1^ Analysis was based on 17,110 any medication records at the month level;

^2^ Analysis was based on 6,688 opioid medication records at the month level;

Models adjusted for Month (March to December), age group (65-69 y, 70-74 y, 75-79 y, 80-84 y, and 85 y+), sex (female, male), socioeconomic status (low, middle, high), regionality (metropolitan and regional areas), and state (New South Wales, NSW; Victoria).

**Supplementary Table 3.** Stratified analyses for the association between demographic characteristics and opioid prescribing status between 2019 and 2020, March to December

|  | 2019 |  | 2020 |  |
| --- | --- | --- | --- | --- |
| **Characteristics** | **Prescribed** | **Odds ratio** | **Prescribed** | **Odds ratio** |
|  | **any opioids^1^** | **(95% CI)** | **any opioids^2^** | **(95% CI)** |
|  | (%) |  | (%) |  |
| **Age group (years)** |  |  |  |  |
| 65-69 | 19 | Ref | 23.7 | Ref |
| 70 – 74 | 18.1 | 0.96 (0.70, 1.30) | 26.4 | 1.19 (0.93, 1.53) |
| 75 – 79 | 20.5 | 1.03 (0.77, 1.37) | 26.7 | 1.184 (0.94, 1.49) |
| 80 – 84 | 20.3 | 0.97 (0.74, 1.28) | 25.0 | 1.07 (0.86, 1.33) |
| 85+ | 22.9 | 1.12 (0.86, 1.45) | 27.8 | 1.18 (0.96, 1.45) |
|  |  |  |  |  |
| **Sex** |  |  |  |  |
| Female | 23.6 | Ref | 28.7 | Ref |
| Male | 17.7 | 0.75 (0.68, 0.83) | 23.2 | 0.80 (0.74, 0.87) |
|  |  |  |  |  |
| **Regionality** |  |  |  |  |
| Metropolitan | 21.1 | Ref | 26.2 | Ref |
| Regional/rural | 24.2 | 1.24 (1.10, 1.40) | 29.70 | 1.57 (1.42, 1.74) |
|  |  |  |  |  |
| **State** |  |  |  |  |
| NSW | 29 | Ref | 40.3 | Ref |
| VIC | 19.4 | 0.57 (0.52, 0.63) | 23.30 | 0.41 (0.38, 0.45) |
|  |  |  |  |  |

^1^ Analysis was based on 34,035 medication records at the month level in 2019;

^2^ Analysis was based on 40,069 medication records at the month level in 2020;

Models adjusted for Month (March to December), age-groups (65-69 y, 70-74 y, 75-79 y, 80-84 y, and 85 y+), sex (female, male), socioeconomic status (low, middle, high), regionality (metropolitan and regional areas), and state (New South Wales, NSW; Victoria).

**Supplementary Table 4.** Stratified analyses for the association between demographic characteristics and opioid prescribing status between the state of New South Wales (NSW) and Victoria from March to December, 2019-2020

|  | NSW | | Victoria |  |
| --- | --- | --- | --- | --- |
| **Characteristics** | **Prescribed** | **Odds ratio** | **Prescribed** | **Odds ratio** |
|  | **any opioids^1^** | **(95% CI)** | **any opioids^2^** | **(95% CI)** |
|  | (%) |  | (%) |  |
| **Year** |  |  |  |  |
| 2019 | 29 | Ref | 19.4 | Ref |
| 2020 | 40.30 | 1.60 (1.49, 1.71) | 23.30 | 1.45 (1.38, 1.53) |
|  |  |  |  |  |
| **Age group (years)** |  |  |  |  |
| 65-69 | 27.20 | Ref | 19.50 | Ref |
| 70 – 74 | 35.50 | 1.48 (0.98, 2.23) | 19.10 | 1.01 (0.79, 1.29) |
| 75 – 79 | 31.70 | 1.47 (1.00, 2.18) | 21.50 | 1.09 (0.86, 1.37) |
| 80 – 84 | 33.30 | 1.46 (1.01, 2.12) | 19.60 | 0.95 (0.76, 1.18) |
| 85+ | 36.10 | 1.50 (1.05, 2.13) | 22.40 | 1.09 (0.88, 1.34) |
|  |  |  |  |  |
| **Sex** |  |  |  |  |
| Female | 37.50 | Ref | 23.00 | Ref |
| Male | 28.80 | 0.78 (0.69, 0.89) | 18.10 | 0.83 (0.77, 0.90) |
|  |  |  |  |  |
| **Regionality** |  |  |  |  |
| Metropolitan | 31.40 | Ref | 21.10 | Ref |
| Regional/rural | 55.10 | 2.36 (1.99, 2.79) | 22.70 | 0.86 (0.78, 0.94) |

^1^ Analysis was based on 17,220 medication records at the month level in NSW;

^2^ Analysis was based on 56,884 medication records at the month level in Victoria;

Models adjusted for Year (2019, 2020), Month (March to December), age group (65-69 y, 70-74 y, 75-79 y, 80-84 y, and 85 y+), sex (female, male), SES (low, middle, high), regionality (metropolitan and regional areas).

**References**

1. Australian Government Department of Health: **MBS Online - Medicare Benefits Schedule**. http://www9.health.gov.au/mbs/fullDisplay.cfm?type=item&q=90020&qt=item. Accessed 18 Jan 2023

2. Australian Government Department of Health: **MBS Online -Medicare Benefits Schedule** 2020. http://www.mbsonline.gov.au/internet/mbsonline/publishing.nsf/Content/news-2020-03-29-latest-news-March. Accessed 18 Jan 2023
